# Case of Tracheotomy

**Published:** 1855-03

**Authors:** J. M. Murdock

**Affiliations:** House Surgeon to Bellevue Hospital


*Case of Tracheotomy.* By J. M. MURDOCK, M. D., House Surgeon to Bellevue Hospital.

The Editors of the New York Medical Times.

GENTLEMEN,—The following case is one in which I have taken much interest; and as it presents a new cause of suffocation, viz., *inspissated mucus in the trachea,* it may not be uninteresting to your readers.

Eliza Campbell, child, aged fourteen months, was admitted into this Hospital on Sunday, Dec. 10th, at 4 o'clock, P.M. The statement of the grandmother, who accompanied the child, was, that she had always been healthy, except the occurrence of a slight convulsion, three months previous, while teething. Since that time she has enjoyed good health, and on the day of the present attack was unusually bright and active; that about an hour previous to her admission, while playing with a piece of meat given her to suck, she was seized with a fit of coughing, and became blue in the face. The child was then taken to a drug-store, and an emetic administered, which produced copious vomiting, but no relief to the dyspnœa. She was then brought to Bellevue Hospital.

When admitted, which was about an hour after the accident, she presented the appearance of being asphyxiated. The lips and face were of a venous hue; respiration was labored and hurried, and the crowing sound of croup was heard, during both inspiration and expiration. Dr. Sands, one of the house physicians, being the first who saw the patient, immediately introduced his finger into the pharynx without detecting any foreign body. He then passed a probang through the œsophagus, with the same result. The patient was failing so fast, that it was the opinion of all the members of the house-staff present, that unless respiration was re-established by an operation, death would soon be the result.

Accordingly, with the assistance of these gentlemen, I performed the operation of tracheotomy, the patient scarcely losing a teaspoonful of blood, and made search for the foreign body, but without success. She was so much relieved by the operation that it was thought proper to discontinue the search until rest was obtained. A silver canula was then introduced into the opening, through which the patient easily respired. She then sank into a quiet slumber, which continued, with slight intermissions, until 12 o'clock, a period of eight hours. At that time, respiration became more labored and hurried, probably from the fact that the foreign body had fallen from the larynx into the trachea, and had stopped at a point below the artificial opening. At 1 o'clock P.M., Drs. J. R. Wood and Sayre, visiting surgeons to the Hospital, saw the patient, and concluded to make further search for the foreign body. Accordingly, Dr. Sayre, by means of a silver probe, after a little search, succeeded in extracting from below the opening a small, hard substance, which, on further examination, was found to be inspissated mucus. After the removal of this foreign body, respiration again became easy, and hopes of permanent recovery were entertained; but in a few hours it became evident that the patient was sinking. The skin was hot; the pulse small, and 190 per minute, the respiration more labored than it had previously been, and 94 per minute. She died at 6 o'clock the next morning, thirty-eight hours after the accident.

On *postmortem* examination, both lungs, with the exception of the middle lobe of the right, presented the appearance of pneumonia in the second stage. This was probably caused by the congestion dependent on imperfect aeration of the blood. No foreign body was found in the larynx, trachea, or bronchi. I think that these appearances justify us in saying, that had the child been brought to the Hospital half an hour earlier, the operation might have preserved life.

*Bellevue Hospital, Dec.* 21, 1854.

